# Attitudes and Awareness Regarding Health Information Sought on Social Media by the Saudi Population: A Cross-Sectional Study

**DOI:** 10.7759/cureus.77759

**Published:** 2025-01-21

**Authors:** Aroob Alhuthail, Bodour Aloraini, Ilal Alhuthail, Sara M Alrashidi, Renad M Alotibi

**Affiliations:** 1 Family Medicine, King Saud University Medical City, Riyadh, SAU; 2 College of Medicine, Imam Mohammad Ibn Saud Islamic University, Riyadh, SAU; 3 College of Medicine, King Saud University, Riyadh, SAU

**Keywords:** awareness, health information, public health, saudi arabia, social media

## Abstract

Background

Social media (SM) platforms are commonly used in Saudi Arabia, even for health information. SM platforms allow users to have conversations, share information, and create web content. Given the growing dependence on social media for health-related concerns, it is critical to understand how Saudis use these platforms to get health information. This study aimed to determine the Saudi population's attitude and awareness regarding health information sought on SM.

Subject and methods

This cross-sectional study was conducted among adults in Riyadh, Kingdom of Saudi Arabia, from September to October 2024. A self-administered questionnaire was distributed randomly in the Medical City King Saud University family medicine clinic. The questionnaire includes socio-demographic data (i.e., age, gender, marital status, etc.), the most commonly used type of SM, and various questions to assess the knowledge and influence of SM on health information.

Results

Among the 330 participants, 117 (63%) were female respondents, and 126 (38.2%) were between 31 and 40 years old. WhatsApp was the most prominent type of SM used at 192 (58.2%). Disease or medical problems were the most notable health information being seen online at 172 (52.1%), and "to be informed" was the most common reason for seeking health information online at 237 (72.4%). The perception of unemployed female respondents that health information obtained from SM is reliable was significantly higher than that of unemployed male respondents (p<0.05). Surprisingly, male participants usually do believe SM can enhance awareness (p = 0.015).

Conclusion

The findings of this study suggest that SM influences the behavior of the adult population seeking health information in Saudi Arabia. Female participants tended to believe that the health information obtained from SM was credible. To be more informed was the primary reason for seeking health information online. There is a need to educate patients visiting family medicine clinics about the reliability of health information obtained online.

## Introduction

Social media (SM) platforms are commonly used in Saudi Arabia, even for health information. Health information is defined as "the data related to a person's medical history, including symptoms, diagnoses, procedures, and outcomes" [1]. The University of South Florida defines social media as "an internet-based form of communication" [2]. SM platforms allow users to have conversations, share information, and create web content" [2]. Given the growing dependence on social media for health-related concerns, it is critical to understand how Saudis use these platforms to get health information. 

Nowadays, people often read or watch videos containing health information, either by actively searching for it or coming across it while browsing social media. On the other hand, some of them like to share their own health experiences, that of their family members, or other people for several reasons. Easy access to information on social media platforms has benefits and harms. It increases the community's health awareness regarding interventions, behavior modification, campaigns, education, surveillance, research, and more [3].

A 2019 study from Riyadh by Marar et al. found that social media usage was widespread in the population, with WhatsApp, YouTube, and Facebook being the most commonly used platforms [4]. However, only a small portion of participants shared medical information online, and many preferred not to disclose their health conditions on social media. The study did not explore the reasons behind sharing medical information or the specific types of health topics people searched for online. 

A study conducted in Boston, USA, by Hausmann et al. (2017) among adolescents and young adults, found that over half of the participants shared health-related content on social media, with mood being the most common topic, followed by prevention, wellness, acute medical conditions, and chronic conditions [5]. Facebook, Instagram, and Twitter (officially X since 2023) were the most popular platforms. Additionally, a 2013 study by Fox and Duggan found that a smaller percentage of people shared health information online, mostly by discussing personal health experiences or asking health-related questions [6]. In contrast, a 2019 study in Kuwait by Alhuwail and Abdulsalam found that YouTube was the most commonly used platform for seeking health information [7]. The most frequent topics searched included medical conditions, treatments, diet, medication, and exercise. Participants mainly sought information to stay informed, out of personal interest, to manage their own health, or to explore alternative treatments. Most believed that online health information influenced their health-related decision-making.

Furthermore, the reliability of health information on social media varied across studies. A 2013 study by Rutsaert et al. found that social media, particularly Facebook and Twitter, is considered one of the least reliable sources of health information [8]. In contrast, a study by Prybutok and Ryan (2015) among 18- to 30-year-old college students found that social media is regarded as a good resource for health information by college students [9].

Understanding patterns of health information consumption on social media in our society will provide insight into how healthcare providers can better communicate with the community, mitigate the spread of misinformation, and support Saudi Arabia’s progress in digital transformation. The role of social media in health education and public health efforts will become more important.

Due to the limited research conducted about social media and health information among the Saudi community, particularly among youth, we propose to explore people's attitudes and awareness regarding health information sought on social media, in addition to identifying the reasons for seeking health information on social media and also to assess the reliability of each social media platform. Furthermore, we intend to assess the association between the reliability with several factors such as age, type of health information, and the source of health information (specialized people posting the information versus nonspecialized people).

## Materials and methods

The Institutional Review Board of King Saud University, College of Medicine, Riyadh, Kingdom of Saudi Arabia, provided ethical approval for this study under Ref. No. 24/1529/IRB, dated September 3, 2024. 

A convenience cross-sectional study was conducted in Riyadh, Kingdom of Saudi Arabia, from September to October 2024. Data was collected through a self-administered questionnaire. Participants were given a self-administered questionnaire with detailed instructions for completing it individually. Questionnaires were distributed randomly in the family medicine clinic as a convenience cross-sectional study design. This study was conducted among 327 Saudi adults in Saudi Arabia (sample size calculated), depending on inclusion and exclusion criteria (Table 1). Participants were required to complete the questionnaire and submit it online. We used a self-designed questionnaire, with face and content validity done (content validity was done by five consultants while face validity was done by 15 persons from the general population, and they found it clear). However, the translation was done twice to Arabic by a medical and non-medical translator, and then they translated back to English.

**Table 1:** Inclusion and exclusion criteria.

Inclusion criteria	Exclusion criteria
Adult	Not living in Saudi Arabia
Using social media	
Follow-up in family medicine clinic	

The participants were informed that their participation in the study was voluntary and confidential, and they were required to complete the questionnaire. Data collection would only take place after the institutional research and ethics committees approved the study. 

Statistical analysis was performed using IBM SPSS Statistics for Windows, Version 25 (IBM Corp., Armonk, NY:). Descriptive statistics (frequencies, percentages, mean, and standard deviation) were used to describe the categorical and quantitative variables. Chi-square tests were used to perform the univariate and multivariate analyses to determine associations with sociodemographic variables and outcomes. We used the formula for a single proportion, assuming a width of 0.05, and a confidence level of 95%. Based on a study conducted in Saudi Arabia, showed that the prevalence of social media users was 85%. With P=0.85, the sample size was calculated at N=327 [4].

## Results

This study enrolled 330 participants. As seen in Table 2, 126 (38.2%) were between 31 and 40 years of age, with female participants being dominant at 208 (63%). Most respondents were Saudi citizens 291 (88.2%) and were married 220 (66.7%). The number of respondents with a bachelor's degree was 179 (54.2%). With regards to occupation, 180 (54.5%) were government employees. The prevalence of participants with a previous history of at least one chronic disease was 104 (31.5%).

**Table 2:** Socio-demographic characteristics of participants (n=330).

Study variables	N (%)
Age group	
18 – 30 years	83 (25.2%)
31 – 40 years	126 (38.2%)
41 – 50 years	73 (22.1%)
>50 years	48 (14.5%)
Gender	
Male	122 (37.0%)
Female	208 (63.0%)
Nationality	
Saudi	291 (88.2%)
Non-Saudi	39 (11.8%)
Marital status	
Single	84 (25.5%)
Married	220 (66.7%)
Divorced	21 (06.4%)
Widowed	05 (01.5%)
Educational level	
Primary and middle School	23 (07.0%)
Secondary school	76 (23.0%)
Bachelor's degree	179 (54.2%)
Higher education	52 (15.8%)
Occupational status	
Unemployed	124 (37.6%)
Government	180 (54.5%)
Private	26 (07.9%)
History of at least one chronic disease	
Yes	104 (31.5%)
No	226 (68.5%)

**Figure 1:**
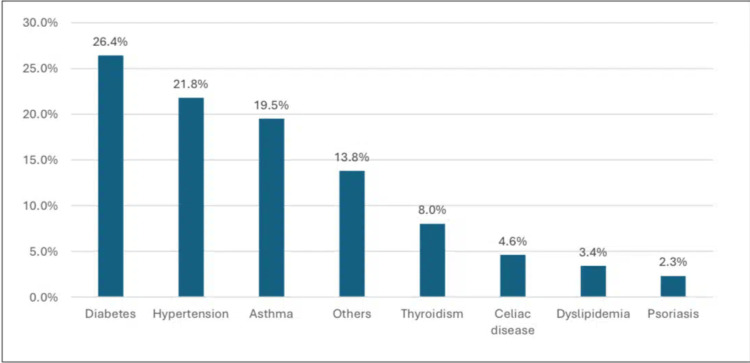
Specific chronic diseases.

In Figure 1, among respondents with a previous history of chronic disease (N = 104), the most common of them was diabetes at 27 (26.4%), followed by hypertension at 22 (21.8%) and asthma at 20 (19.5%).

**Figure 2:**
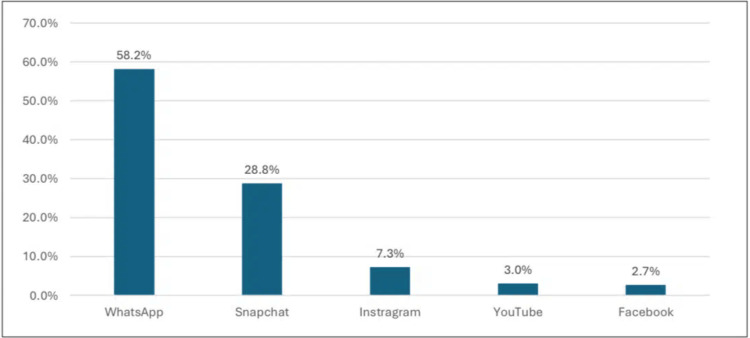
Frequently used types of social media.

Figure 2 depicts that the most commonly used type of social media was WhatsApp at 192 (58.2%), followed by Snapchat at 95 (28.8%), and Instagram at 24 (7.3%).

**Figure 3:**
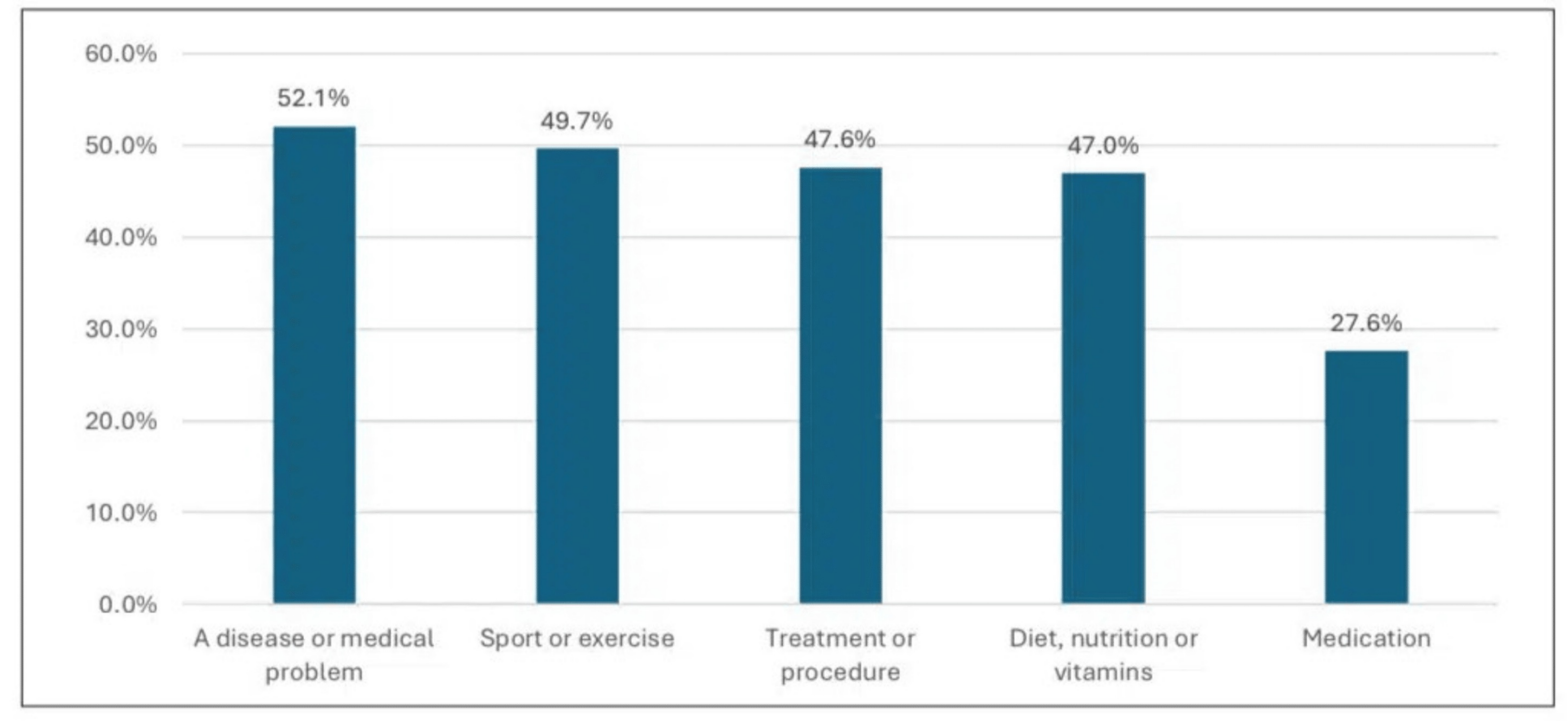
Types of health information seen online.

Figure 3 illustrates that the most common types of health information seen online were a disease or medical problem at 171 (52.1%), followed by sport or exercise at 164 (49.7%), and treatment or procedure at 157 (47.6%).

**Figure 4:**
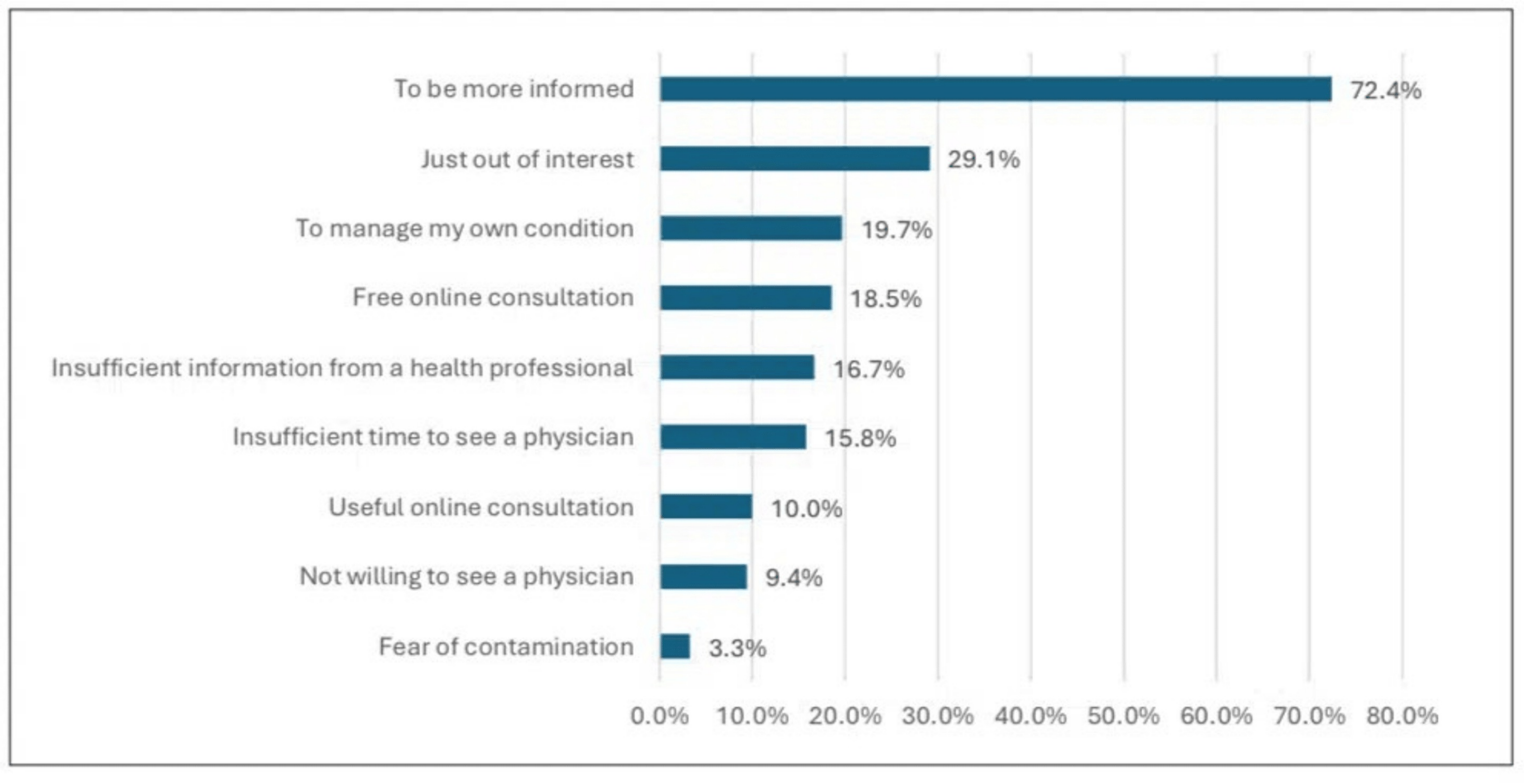
Reasons for seeking health information online before visiting the physician online.

Figure 4 shows that the most prominent reason for seeking health information online before visiting the physician was to be more informed, which stood at 239 (72.4%), followed by interest at 95 (29.1%), and to manage own condition at 66 (19.7%).

Regarding the assessment and influence of SM on health information, as seen in Table 3, participants who believe that health information on SM influences health status, the specific doctor, and the specific hospital were at 137 (41.5%), 184 (55.6%), and 190 (57.6%), respectively. More than half, 170 (51.5%), believe that health information obtained from SM is reliable. More than three-quarters, 248 (75.2%), believe that SM enhances awareness. Approximately 137 (41.5%) indicated that they sometimes see medical information on SM. Respondents who use search engines for health information on a monthly basis constitute 87 (26.4%). Nearly all 264 (80%) searched how reliable the new information reads from SM. Most of the respondents, 242 (73.3%), preferred search engines for searching health information. It was observed that female participants were more associated with the belief that health obtained from SM was reliable (p = 0.034), while male participants have been shown to be more disbelieving in the potential of SM to enhance (p = 0.015) awareness.

**Table 3:** Knowledge and influence of SM on health information in relation to gender. § P-value has been calculated using the Chi-square test; ** Significant at p<0.05 level. SM: social media.

Variables	Overall N (%) ^(n=330)^	Gender		χ2	P-value^§^
		Male N (%) ^(n=122)^	Female N (%) ^(n=208)^		
Does health information on SM influence your health status?					
Yes	137 (41.5%)	60 (49.2%)	77 (37.0%)	5.310	0.070
No	152 (46.1%)	51 (41.8%)	101 (48.6%)		
I don't know	41 (12.4%)	11 (09.0%)	30 (14.4%)		
Does SM influence you to select a specific doctor?					
Yes	184 (55.8%)	63 (51.6%)	121 (58.2%)	1.333	0.514
No	121 (36.7%)	49 (40.2%)	72 (34.6%)		
I don't know	25 (07.6%)	10 (08.2%)	15 (07.2%)		
Does SM influence you to select a specific hospital?					
Yes	190 (57.6%)	72 (59.0%)	118 (56.7%)	0.215	0.898
No	122 (37.0%)	44 (36.1%)	78 (37.5%)		
I don't know	18 (05.5%)	06 (04.9%)	12 (05.8%)		
In your opinion, how reliable is health information obtained through SM/search engines?					
Very reliable	13 (03.9%)	09 (07.4%)	04 (01.9%)	6.750	0.034**
Reliable	170 (51.5%)	57 (46.7%)	113 (54.3%)		
Unreliable	147 (44.5%)	56 (45.9%)	91 (43.8%)		
Do you believe that SM enhances awareness?					
Yes	248 (75.2%)	89 (73.0%)	159 (76.4%)	8.392	0.015**
No	37 (11.2%)	21 (17.2%)	16 (07.7%)		
I don't know	45 (13.6%)	12 (09.8%)	33 (15.9%)		
How frequently do you see medical information on SM?					
Very often	45 (13.6%)	16 (13.1%)	29 (13.9%)	1.367	0.850
Often	124 (37.6%)	42 (34.4%)	82 (39.4%)		
Sometimes	137 (41.5%)	54 (44.3%)	83 (39.9%)		
Rarely	18 (05.5%)	07 (05.7%)	11 (05.3%)		
Never	06 (01.8%)	03 (02.5%)	03 (01.4%)		
The frequency of using search engines for health information					
Daily	59 (17.9%)	19 (15.6%)	40 (19.2%)	3.724	0.590
Weekly	78 (23.6%)	25 (20.5%)	53 (25.5%)		
Monthly	87 (26.4%)	34 (27.9%)	53 (25.5%)		
Annually	17 (05.2%)	09 (07.4%)	08 (03.8%)		
Rarely	87 (26.4%)	34 (27.9%)	53 (25.5%)		
Never	02 (0.60%)	01 (0.80%)	01 (0.50%)		
When reading new health information on SM, do you search for how reliable it is?					
Yes	264 (80.0%)	97 (79.5%)	167 (80.3%)	0.029	0.864
No	66 (20.0%)	25 (20.5%)	41 (19.7%)		
Do you prefer to use search engines or SM?					
Search engine	242 (73.3%)	94 (77.0%)	148 (71.2%)	1.367	0.242
Social media	88 (26.7%)	28 (23.0%)	60 (28.8%)		

Continuing with the assessment and influence of SM on health information, as seen in Table 4 respondents who sometimes use the Internet for health conditions before visiting the physician constitute 159 (48.2%). Participants who were influenced by the Internet or social media in taking specific medication were 137 (41.5%). Only 100 (30.3%) share medical experience with the public through SM. The most common topic shared through SM was wellness and prevention, by 205 (62.1%) participants, while the most common SM app for sharing posts was WhatsApp, by 164 (49.7%) participants. Approximately 82 (24.8%) rarely watch an influencer who shares an advertisement for medication on SM, while 106 (32.1%) daily watch an influencer who shares advertising for plastic procedures/surgery. When comparing males and females, it was observed that female participants were more likely to believe that health obtained from SM was reliable (p=0.034), that they were more associated with sharing posts through SM about wellness and prevention (p=0.039), and were more frequently seeing posts from influencers regarding plastic surgery (p=0.016). Male participants were associated with being doubtful that SM could enhance awareness (p=0.015).

**Table 4:** Knowledge and influence of SM on health information in relation to gender (cont'd.). * Variable with multiple response answers; § P-value has been calculated using the Chi-square test; ** Significant at p<0.05 level. SM: social media.

Variables	Overall N (%) ^(n=330)^	Gender		χ2	P-value^§^
		Male N (%) ^(n=122)^	Female N (%) ^(n=208)^		
Searching the Internet for health conditions before visiting the physician					
Very often	34 (10.3%)	12 (09.8%)	22 (10.6%)	2.938	0.401
Often	87 (26.4%)	26 (21.3%)	61 (29.3%)		
Sometimes	159 (48.2%)	63 (51.6%)	96 (46.2%)		
Rarely	50 (15.2%)	21 (17.2%)	29 (13.9%)		
Does the Internet or SM influence how you use a specific medication?					
Yes	137 (41.5%)	50 (41.0%)	87 (41.8%)	0.023	0.881
No	193 (58.5%)	72 (59.0%)	121 (58.2%)		
Sharing medical experience with the public via SM					
Yes	100 (30.3%)	44 (36.1%)	56 (26.9%)	3.043	0.081
No	230 (69.7%)	78 (63.9%)	152 (73.1%)		
Your health posts on SM usually involve which of the following online*					
Mood	110 (33.3%)	39 (32.0%)	71 (34.1%)	0.163	0.687
Wellness and prevention	205 (62.1%)	67 (54.9%)	138 (66.3%)	4.268	0.039**
Acute medical condition	40 (12.1%)	17 (13.9%)	23 (11.1%)	0.597	0.440
Chronic medical conditions	56 (17.0%)	17 (13.9%)	39 (18.8%)	1.266	0.261
I don't share	32 (09.7%)	14 (11.5%)	18 (08.7%)	0.699	0.403
Evaluating a physician/hospital via SM					
Yes	118 (35.8%)	43 (35.2%)	75 (36.1%)	0.664	0.717
No	174 (52.7%)	67 (54.9%)	107 (51.4%)		
I don't know	38 (11.5%)	12 (09.8%)	26 (12.5%)		
On which site do you usually share the post?					
WhatsApp	164 (49.7%)	65 (53.3%)	99 (47.6%)	6.378	0.271
Snapchat	54 (16.4%)	16 (13.1%)	38 (18.3%)		
Instagram	16 (04.8%)	03 (02.5%)	13 (06.3%)		
Facebook	06 (01.8%)	04 (03.3%)	02 (01.0%)		
YouTube	06 (01.8%)	02 (01.6%)	04 (01.9%)		
I don't share	84 (25.5%)	32 (26.2%)	52 (25.0%)		
How frequently do you see an influencer advertising a medication on SM?					
Daily	69 (20.9%)	23 (18.9%)	46 (22.1%)	4.783	0.443
Weekly	78 (23.6%)	33 (27.0%)	45 (21.6%)		
Monthy	65 (19.7%)	18 (14.8%)	47 (22.6%)		
Annually	13 (03.9%)	06 (04.9%)	07 (03.4%)		
Rarely	82 (24.8%)	32 (26.2%)	50 (24.0%)		
Never	23 (07.0%)	10 (08.2%)	13 (06.3%)		
How frequently do you see an influencer advertising plastic procedures/surgery on SM?					
Daily	106 (32.1%)	25 (20.5%)	81 (38.9%)	13.917	0.016**
Weekly	75 (22.7%)	28 (23.0%)	47 (22.6%)		
Monthy	65 (19.7%)	29 (23.8%)	36 (17.3%)		
Annually	12 (03.6%)	06 (04.9%)	06 (02.9%)		
Rarely	47 (14.2%)	22 (18.0%)	25 (12.0%)		
Never	25 (07.6%)	12 (09.8%)	13 (06.3%)		

Exploring the relationship between the awareness of the reliability of health information obtained on SM among the socio-demographic characteristics and the most commonly used SM app, as seen in Table 5, found that unemployed participants were more likely to believe that the health information obtained from SM was reliable (p = 0.035). No significant relationships were observed between the awareness of the reliability of health information on SM in terms of age, gender, nationality, marital status, educational level, history of at least one chronic disease, and the type of SM being used (p > 0.05).

**Table 5:** Relationship between the awareness of the reliability of health information on SM among the socio-demographic characteristics and the most commonly used SM apps (n=330). § P-value has been calculated using the Chi-square test; ** Significant at p<0.05 level. SM: social media.

Factor	Awareness of the reliability of health information on SM		χ2	P-value^§^
	Unreliable N (%) ^(n=147)^	Reliable N (%) ^(n=183)^		
Age group				
≤40 years	92 (62.6%)	117 (63.9%)	0.064	0.800
>40 years	55 (37.4%)	66 (36.1%)		
Gender				
Male	56 (38.1%)	66 (36.1%)	0.144	0.704
Female	91 (61.9%)	117 (63.9%)		
Nationality				
Saudi	132 (89.8%)	159 (86.9%)	0.663	0.416
Non-Saudi	15 (10.2%)	24 (13.1%)		
Marital status				
Unmarried	35 (23.8%)	49 (26.8%)	0.378	0.539
Married	112 (76.2%)	134 (73.2%)		
Educational level				
Secondary school or below	42 (28.6%)	57 (31.1%)	0.258	0.612
Bachelor's degree or higher	105 (71.4%)	126 (68.9%)		
Occupational status				
Unemployed	46 (31.3%)	78 (42.6%)	4.462	0.035**
Employed	101 (68.7%)	105 (57.4%)		
History of at least one chronic disease				
Yes	48 (32.7%)	56 (30.6%)	0.159	0.690
No	99 (67.3%)	127 (69.4%)		
Type of SM				
WhatsApp	85 (57.8%)	107 (58.5%)	0.014	0.906
Non-WhatsApp	62 (42.2%)	76 (41.5%)		

## Discussion

SM influence on health-seeking information

This study revealed that around half of our sample population was influenced by SM regarding their health condition, the use of specific medication, and the choice of doctor and hospital; these influences, however, did not differ significantly between male and female individuals (p > 0.05). This is consistent with the study of Althukair et al. (2021) [10]. Based on the reports, SM used for health-seeking information among Saudi participants was common, and female participants tended to use SM to contact their physicians. Other demographic characteristics, such as age and education, were also significantly associated with SM use for obtaining health-related information [10]. This corroborates the study of Wijayanti et al. (2022) [11]. Respondents believed that the perceived benefits, health self-efficacy, and subjective norms motivated the intention to seek health information on SM. Additionally, female participants perceived risk greatly affected the intention to seek health information, but it did not influence the intention to seek health information in the overall population and the male group [11]. However, a lower reliance on health information given by social media has been reported by Achampong et al. (2020) [12]. Only 64 (18.1%) responded that they had ever been influenced by the health information posted on social media, and only 39 (11%) were positively influenced by social media to stop medication or treatment [12]. The differences in the population group, regional settings, and study design contributed to the conflicting outcomes. Hence, further studies are necessary to establish SM's influence on health-seeking information behavior in the region. 

Reliability of health information from SM

More than half of the respondents were of the opinion that health information acquired from SM was reliable. Supporting their opinions, a vast majority of 264 (80%) searched how reliable the new health information they read online was. The search engine was the most commonly preferred method for finding new health information online, and over one-quarter of the population often searched the Internet for medical conditions before visiting physicians. Furthermore, unemployed participants were more likely to believe that health information received from SM was reliable, but employed participants believed otherwise. However, our results showed no significant link between the awareness of the reliability of health on SM in terms of age, nationality, marital status, education, and history of at least one chronic disease (p > 0.05). This is not in agreement with the study of Gong and Verboord (2020) [13]. Findings show that younger subjects with lower education, watching television, using SM platforms (WeChat, Qzone) more often, and reading newspapers were significantly more likely to consider online information credible [13]. On the contrary, a previous study conducted during COVID-19 found that nearly two-thirds of the population was not inclined to fact-check what they read online, despite the fact that there was a high level of misinformation regarding the accuracy of the COVID-19-related information on SM. Nevertheless, there are greater odds of getting vaccinated among those who subscribed to more credible scientific resources on SM at the peak time of the pandemic [14]. It is important to cross-check the credibility of health information seen on SM. Misinformation read from SM can be harmful, which could lead to a worst-case scenario in terms of health outcomes.

Preferred SM platforms for health information

Among adult subjects living in Saudi Arabia indicated that the most commonly used type of SM was WhatsApp 192 (58.2%) and Snapchat (95, 28.8%). Other SM platforms were less rated, such as Instagram 24 (7.3%), YouTube 10 (3%), and Facebook 8 (2.7%). The use of SM apps for health-related information varies according to region. For example, YouTube was the most commonly used SM app in Kuwait for help-seeking information [7], while in Ghana, the type of SM app was almost consistent with our results, with WhatsApp and Facebook use being dominant [12]. However, in China, the use of WeChat, Qzone, and Baidu Tieba was common among the Chinese population [13]. Our results were consistent with the use of the most common SM apps among the Saudi Arabian population [4,10,15,16].

Reasons for seeking health information

This study noted that getting more information about the medical condition was the most prominent reason for seeking health information online. Other reasons were less prominent but are still invaluable, including self-interest in certain topics, the treatment of one's condition, lack of information received from a health professional, lack of time to see a physician, useful online consultation, unwillingness to see a physician, and fear of contamination. Further, the most frequent type of health information seen online was about the disease or medical condition, followed by sport or exercise, treatment or procedures, and diet, nutrition, or vitamins, while the topic of medication was less rated. Consistent with our reports, among Kuwaiti residents, general curiosity about the topic, such as being more informed and just out of interest, was cited as the most common reason for online seeking health information, while disagreement with a health professional's advice and limited time with a healthcare provider was recognized as the least cited reasons [7]. Similarly, another study conducted in Saudi Arabia, consistent with our findings, showed that online health-related information seekers most frequently searched for nutrition and diet details (241, 62.6%), followed by medication and drug side effects (181, 47%), and fitness and exercise information (149, 38.7%) [17]. Among Jeddah's residents, the most common reason was that they did not want to visit the hospital. However, 533 (42.3%) said they tried treatment for someone with a similar experience or illness through SM, and 460 (35.4%) reported searching for health-related information on the Internet monthly [15]. In our study, however, approximately 26.7% frequently read medical information on SM, and 242 (73.3%) used search engines for health-related information online, consistent with previous reports.

Sharing health information

Sharing health information online is vital to raising public awareness about emerging health concerns. In this study, 30.3% of our population shared their medical experience with the public using social media, with wellness and prevention being the most common health information posted online, and female individuals were more likely to share this type of medical experience (p = 0.039). Other health-related topics being shared were mood and acute and chronic medical conditions. In addition, approximately 21% of our subjects regularly saw an influencer sharing an advertisement for medication, and about one-third saw an influencer sharing an advertisement about plastic procedure surgery on SM, which was prevalent in female subjects (p = 0.016). These findings are almost consistent with the study of Hausmann et al. (2017), suggesting that 105 (51.5%) had shared health information on SM, where mood, wellness, and acute medical conditions were the most common topics being shared online [5]. Adding that participants with perceived poor health tended to share health-related information than the other subject groups [5]. This corroborates the reports of Achampong et al. (2020) [12]. Nearly half indicated that they shared health-related information online. However, only 39 (19.5%) had the systems to verify the authenticity of what they shared online [12].

This study explores the awareness of the Saudi adult population regarding health information sought on SM and determines the most common reasons for seeking health information. The findings of this study will be a great addition to the literature, given the widespread use of SM, which influences all walks of life, including health-seeking information.

Study limitations

The findings of this study accounted for several limitations. First, this study uses subjective questionnaires to measure participants' perceptions of SM, which could potentially be the source of some biases, including response styles, overconfidence, and casting doubts on the validity of the measured constructs. Second, the convenience sampling method could result in sampling bias and could lead to a lack of diversity in the target population. Lastly, a cross-sectional survey could be prone to bias, unable to determine cause and effect, and cannot be used to measure behavior over time.

## Conclusions

This study supports the literature that social media influences health-seeking information behavior. Further, more than half of the participants were of the opinion that health information acquired from SM is reliable. However, employed male participants tended to be independent of the health information given by SM and did not believe that it could enhance awareness either. Female participants who shared information on SM were more likely to share wellness and prevention than either mood or medical conditions. The most dominant reason for seeking health information online was to acquire more information about the condition. Following literature across the region, this study also found that WhatsApp was the most commonly used SM app for health-seeking information among the adult population in Saudi Arabia. There is a need to educate the public regarding SM's quality of health information. Healthcare authorities had the vital role of educating the public regarding the accuracy and reliability of online health information. Promoting appropriate websites (i.e., government websites, WHO, etc.) could reduce the risk of misleading information among the population.
